# Effect of NaAlO_2_ Co-Electrical Parameters on the Wear Resistance of MAO/MoS_2_ Self-Lubricating Composite Coatings

**DOI:** 10.3390/ma18081825

**Published:** 2025-04-16

**Authors:** Feiyan Liang, Erhui Yang, Na Jia, Weizhou Li, Xiaolian Zhao, Ruixia Yang

**Affiliations:** 1 School of Resources, Environment and Materials, Guangxi University, Nanning 530004, China; 19977470215@163.com (F.L.); yangeh89@163.com (E.Y.); 18071394431@163.com (N.J.); wz-li@hotmail.com (W.L.); xiaolianbsh@126.com (X.Z.); 2 Key Laboratory of Beibu Gulf Offshore Engineering Equipment and Technology, Education Department of Guangxi Zhuang Autonomous Region, Beibu Gulf University, Qinzhou 535011, China; 3 School of Materials Science and Engineering, Xiamen University of Technology, Xiamen 361024, China

**Keywords:** TC4 titanium alloy, orthogonal experiment, hydrothermal synthesis, self-lubricating, wear resistance

## Abstract

This study aims to enhance the wear resistance of MAO/MoS_2_ composite coatings fabricated on TC4 titanium alloy substrates through a composite process of microarc oxidation (MAO) and hydrothermal synthesis. The MAO treatment experiments were designed according to the L16 (4^5^) orthogonal array to optimize the NaAlO_2_ concentration and electrical parameters (oxidation voltage, frequency, duty ratio, and treating time), with four levels for each factor. The optimized MAO process parameters were identified as a NaAlO_2_ concentration of 10 g/L, an oxidation voltage of 500 V, a frequency of 200 Hz, a duty ratio of 20%, and a treating time of 30 min. The experimental results indicated a notable reduction in porosity, from 4.45% to 0.30%, in the optimized composite coating. Concurrently, there was a 43.2% increase in microhardness and a 327.9% increase in adhesive strength. Furthermore, the average coefficient of friction (CoF) of the composite coating was observed to be 0.13 at a high load of 20 N and a wear time of 20 min, representing a significant reduction of 68.5% compared to the CoF of the single MAO coating.

## 1. Introduction

The distinctive properties of titanium alloys, including high specific strength, exceptional corrosion resistance, and high-temperature oxidation resistance, are fully utilized in a multitude of fields, including aerospace, defense, marine engineering, chemical processing, and biomedical materials [1,2,3,4]. Indeed, the wear resistance of titanium alloys is inadequate [5,6], as evidenced by their low hardness, which renders them susceptible to adhesive wear. Consequently, the provision of effective lubrication to moving sub-structural components represents a significant challenge, resulting in wear failure occurring in severe friction environments. This markedly diminishes the lifespan of the components and constrains their applications [7]. A substantial corpus of research has been conducted domestically and abroad on surface treatment technology with the objective of preparing coatings with superior friction and wear properties on the surface of titanium alloys, with the aim of enhancing their wear resistance [7,8,9].

Micro-arc oxidation (MAO) is a high-pressure plasma electrolytic oxidation [10] process that provides a high-energy-density surface treatment of titanium alloys [11,12]. This process generates insulating and wear-resistant hard oxide ceramic coatings in situ, thereby significantly optimizing the surface microhardness, wear resistance, and service life of titanium alloys [13]. The advantages of MAO treatment of titanium alloys include a simple process, environmental protection, the absence of toxic or hazardous gas emissions, high coating hardness, and superior wear resistance compared to the substrate [14]. However, the elevated and fluctuating coefficient of friction (CoF) at micropores and microcracks of MAO coatings on titanium alloys has been observed to exacerbate wear failure [15,16,17] and fail to adapt to frictional wear in more severe friction environments [18]. Two main approaches are typically employed to address the issue of loose and porous structural defects in MAO coatings: doping nanoparticles or depositing them into self-lubricating coatings [19]. This not only effectively enhances the tribological properties of the coatings but also optimally leverages the benefits of microporous and microcracked storage lubrication materials.

Consequently, the significant research focus has shifted to the introduction of suitable lubricating phases into MAO coatings of titanium alloys, with the objective of obtaining a self-lubricating function [20,21]. This kind of self-lubricating modification technology can be classified into two categories: direct composite technology and secondary composite technology. In the former approach, functional particles are introduced directly into the electrolyte and subsequently combined through a series of processes, including electrophoresis, diffusion, and adsorption [21]. Ma et al. [22] introduced Molybdenum disulfide (MoS_2_) into the electrolyte and conducted electrophoresis to create a composite film, resulting in a 35% reduction in CoF, a 95% reduction in wear loss, and a notable enhancement in tribological performance when compared to the ZL109 titanium alloy substrate. Mu et al. [23] added solid-phase particles of MoS_2_ to the electrolyte and compounded the film on the surface of TC4 titanium alloy, demonstrating a CoF of 0.12 and exhibiting promising self-lubricating properties. The self-lubricating composite coatings prepared by Aliasghari et al. [24] with the direct addition of PTFE particles demonstrated average wear resistance with only a slight reduction in CoF. However, the principal limitation of direct composite technology is the quantity of functional particles incorporated and the unequal dispersion of film formation resulting from particle agglomeration and sedimentation. In addition to MAO treatment, other surface modification techniques, including dip-coating, electrophoretic deposition, magnetron sputtering, and others, have been employed in the context of secondary composite technology. The incorporation of a self-lubricating layer and MAO coating composite markedly enhances tribological performance. Martini et al. [25] utilized an aerosol spray technique to deposit polytetrafluoroethylene (PTFE) onto the surface of a TC4/MAO coating, thereby forming a continuous and uniform self-lubricating layer with a CoF of only 0.2 at low loads. Cheng et al. [26] prepared CrN coatings on the surface of TC4/MAO coatings by the multi-arc ion plating (MAIP) technique, resulting in a reduction in surface roughness and a decrease in COF to 0.2. Li et al. [27] employed the magnetron sputtering process to deposit a DLC film on the surface of a Ti/MAO coating. The resulting composite coating exhibited a COF of 0.24 in the stable friction stage, as well as a notable reduction in surface roughness and wear rate. The tribological behavior of the coatings prepared by secondary composite technology was excellent; however, the challenges associated with the cumbersome operation and low filling efficiency remain to be addressed.

The hydrothermal composite method represents an appropriate choice for the preparation of self-lubricating composite coatings. This method offers several advantages, including simple operation, low cost, high product purity, excellent anti-wear properties, and a self-lubricating effect. Molybdenum disulfide (MoS_2_), also known as the “king of lubrication”, exhibits favorable mechanical properties and remarkable friction reduction, thereby demonstrating its efficacy in solid lubrication and the enhancement of tribological properties [19]. Tuo et al. [28] synthesized both MAO and MoS_2_ coatings in situ simultaneously on Al alloy substrates, resulting in a notable reduction in CoF by 80%. Li et al. [29] prepared YSZ-MoS_2_ composite coatings on steel substrates, achieving a CoF of less than 0.1. Lv et al. [30] fabricated MAO/MoS_2_ composite coatings on 6063 Al alloy substrates, maintaining a CoF of approximately 0.2. It can be concluded that the hydrothermal compounding method has the potential to enhance the tribological properties of the substrate in a cost-effective and efficient manner. This study plans to design an orthogonal experiment for efficiently determining the optimal process of MAO, with the objective of optimizing maximizely the wear resistance of the after-composite coating while concomitantly minimizing the number of required experiments [31]. Inspired by various existing literatures, in this study, an orthogonal experiment was conducted on TC4 titanium alloy substrates with NaAlO_2_ concentration and electrical parameters as variables. Based on the result, hydrothermal synthesis was performed to fabricate MAO/MoS_2_ self-lubricating composite coatings. This is the inaugural investigation into the impact of NaAlO_2_ co-electrical parameters on the tribological properties of MAO/MoS_2_ composite coatings prepared on TC4 titanium alloy substrates. The central objective of this experiment was to explore the effect of process parameters of MAO on the wear resistance of composite coatings.

## 2. Experimental

### 2.1. Fabrication of MAO Coatings

The substrate is TC4 with dimensions of 20 × 15 × 1.5 mm^3^. Prior to MAO, the substrates were ground with three grades of silicon carbide paper (400, 1000, and 1500#) in turn, followed by being ultrasonically washed with ethanol for 10 min, and dried with cool air. Given that the MAO layer prepared in constant voltage mode exhibits more uniform pore size and distribution [32,33,34], Wang et al. [35] have found that the MAO layer synthesized in the aluminate electrolyte has the least degree of influence on the MoS_2_ synthesized hydrothermally. Subsequently, it can be concluded that the constant voltage mode and the aluminate electrolyte system are optimal for the MAO treatment in this experiment.

A domestic pulse power supply (Jinan Nenghua Electromechanical Equipment Co., Ltd., Jinan, China) was employed for MAO treatment in constant voltage mode. The aluminate electrolyte formulation comprised 10, 15, 20, or 25 g/L NaAlO_2_ (Tianjin Damao Chemical Reagent Factory, Tianjin, China) and 1 g/L NaOH (Shanghai Macklin Biochemical Co., Ltd., Shanghai, China). Blank TC4 alloy underwent oxidation for 15, 20, 25, or 30 min at a positive voltage control of 450, 475, 500, or 525 V, a negative voltage of 50 V, a frequency of 200, 300, 400, or 500 Hz, and a duty ratio of 5, 10, 15, or 20%. A stirring and cooling system was employed throughout the experiment to ensure the temperature of the electrolyte remained below 50 °C.

In order to investigate the effect of NaAlO_2_ co-electrical parameters on the wear resistance of composite coatings and optimize the parameters, L16(4^5^) orthogonal tests were conducted with five factors: NaAlO_2_ concentration, oxidation voltage, current frequency, duty ratio, and treating time. Each factor was selected at four levels. Factors and levels of the orthogonal experiments are presented in Table 1. Following MAO treatment, the specimens were rinsed with deionized water and subsequently cleaned ultrasonically in ethanol for 10 min. They were then dried at room temperature and stored.

### 2.2. Fabrication of MAO/MoS_2_ Self-Lubricating Composite Coatings

The synthesis of MoS_2_ self-lubricating layers was based on the process parameters and operating methods in hydrothermal synthesis experiments by Lv et al. [30] and Liu et al. [36]. The MAO coatings, which were prepared from the orthogonal experimental array shown in Table 2, were utilized as the substrate, and the MoS_2_ coating was synthesized in situ via the hydrothermal method in a blast drying oven and compounded into an MAO/MoS_2_ self-lubricating coating, whose coating designation presented in Table 2. In the hydrothermal reaction solution, sodium molybdate (Na_2_MoO_4_, Sinopharm Chemical Reagent Co., Ltd., Shanghai, China) was used as the molybdenum source, and thiourea (CH_4_N_2_S, Guangdong Guanghua Sci-Tech Co., Ltd., Shantou, China) as the sulfur source. The hydrothermal process parameters were as follows: a 1:4 atomic ratio of Mo to S, a temperature of 220 °C, a hydrothermal time of 24 h, and a reactor filling degree of 70%. Prior to the hydrothermal synthesis, the reaction solution was subjected to 30 min of magnetic stirring to ensure thorough mixing. Following immersion of the MAO specimen, it was subjected to ultrasonication for 10 min. Thereafter, impregnation was conducted under a vacuum pressure of −0.08 MPa (the corresponding vacuum degree is 10^4^ Pa, which belongs to a rough vacuum) for 1 h. This process was employed to ensure that the solution penetrated into the micropores and cracks on the MAO coating substrates. Subsequently, the samples were transferred to a para-polyphenylene (PPL) liner with a capacity of 100 mL, and the hydrothermal reactor (kettle body made of 304 stainless steel) was assembled and placed in a blast drying oven for hydrothermal synthesis. Once the reaction was complete, the specimen was cooled in the oven, removed, rinsed three times with deionized water, dried at room temperature, and set aside.

### 2.3. Characterization of Coating Properties

The phase compositions of MAO coatings and composite coatings were analyzed by X-ray diffraction (XRD, Rigaku D/MAX, Rigaku, Tokyo, Japan) with the following parameter settings: a Cu-Kα ray source, an operating voltage of 40 kV, an operating current of 100 mA, a scanning range of 10–85°, and a scanning speed of 10°/min. Following the completion of the test, a qualitative analysis of the crystal structures was conducted in comparison to the JCPDS cards. The microscopic morphology of the coating surface and cross-section was observed using a scanning electron microscope equipped with an energy-dispersive X-ray spectrometer (SEM/EDS, TM 4000PLUS, Hitachi, Tokyo, Japan). The Image J 1.54i software was employed for the analysis of the porosity and thickness of the coatings.

Enhancing the hardness and adhesive strength of the coating can improve its wear resistance significantly [37]. The increase in hardness was demonstrated to affect substantially on friction and wear [38], thereby enhancing the friction-reducing performance of the coating. Consequently, the microhardness, adhesive strength, and CoF of the composite coatings were used to evaluate their anti-wear and friction reduction properties. A Vickers hardness tester (HVS-30, Shanghai Zhongyan Instrument Manufacturing Co., Ltd., Shanghai, China) was employed to ascertain the microhardness of the specimens. Five distinct areas were selected on the surface of each specimen, and the microhardness was determined using a force of 49 N and a set time of 5 s. Specific tests were conducted in accordance with the Vickers hardness test methods delineated in ISO 6507-1:2018 [39]. The automatic scratch tester (WS-2005, Lanzhou Zhongke Kaihua Technology Co., Ltd., Lanzhou, China) was employed to conduct the dynamic load scratch experiment, with the critical load utilized to assess the coating and substrate adhesion strength. The parameters for each specimen were set to 120 N of loading load, 100 N/min of loading rate, and 5 mm of scratch length. The scratch tests referenced the test standards of ISO 2409:2013 [40] and ASTM D3359-21 [41]. The CoF of the coatings and GCr15 steel balls grinding against were determined by a ball-and-disk friction tester (HT-1000, Lanzhou Zhongke Kaihua Technology Co., Ltd., Lanzhou, China). The load was set to 20 N, the fixed rotation speed was 224 rpm-min^−1^, the rotation time was 20 min for the MAO samples and 90 min for the composite samples, and the diameter of the rotation track was 4.0 mm. For specific friction and wear tests, refer to the test methods detailed in ASTM G99-23 [42].

## 3. Results

### 3.1. Orthogonal Experiment Results

**Figure 1:**
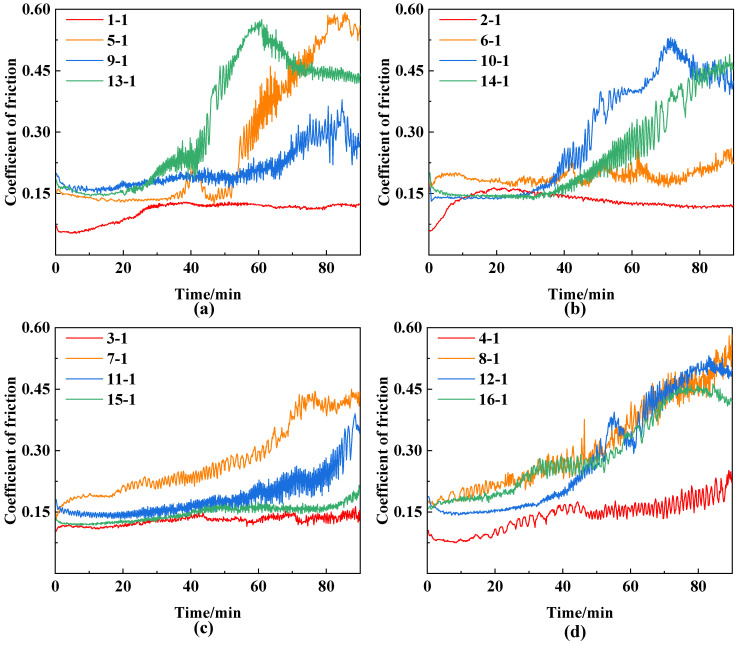
Friction coefficient plots of composite coatings at varying oxidation voltages: (**a**) 450 V, (**b**) 475 V, (**c**) 500 V, (**d**) 525 V.

The array and experimental results of the orthogonal experiment are presented in Table 3. The average CoF (obtained from Figure 1), microhardness, and adhesive strength of the composite-coated specimens are utilized as indicators of the wear resistance of the MAO/MoS_2_ composite coatings. Table 3 reveals that the average CoF of the 13-1 and 1-1 specimens corresponds to the highest value of 0.33 and the lowest value of 0.11, respectively. Similarly, the highest value of microhardness, 8.75 GPa, and the lowest value, 7.85 GPa, are achieved by the 9-1 and 10-1 specimens, respectively. Additionally, the highest value of adhesive strength is 106.43 N for the 1-1 specimen, while the lowest is 47.30 N for the 5-1 specimen.

In Table 3, *k _i_* (*i* = 1, 2, 3, 4) represents the mean value of the corresponding level “*i*” for each factor, representing the average of the experimental results at that level. Meanwhile, R stands for the extreme deviation of *k _i_* (*i* = 1, 2, 3, 4), which serves as a measure of the overall effect of the factors on the results of the experiment. As demonstrated in Table 3, the order of influence of the factors on the average CoF of composite coatings is as follows: NaAlO_2_ concentration > voltage > duty radio > frequency > treating time. The maximum value of R is 0.14, and the minimum value is 0.14 for factors A and E, respectively. Furthermore, the values of *k _1_* for factor “A”, the *k _3_* for factor “B”, *k _1_* for factor “C”, *k _4_* for factor “D”, and *k _4_* for factor “E” are 0.13, 0.19, 0.19, 0.17, and 0.21, respectively. These values correspond to the lowest mean CoF observed among the four levels of each factor. Therefore, the optimal solution is A_1_B_3_C_1_D_4_E_4_.

Secondly, the order of influence for microhardness is determined to be duty radio > voltage > frequency > NaAlO_2_ concentration > treating time. The maximum value of R 0.48 GPa and the minimum value of R 0.10 GPa are found to correspond to factors “D” and “E”, respectively. Moreover, the highest values of average microhardness among the four levels under each factor are identified as follows: The values of *k _1_* for factor “A”, *k _3_* for factor “B”, *k _1_* for factor “C”, *k _4_* for factor “D” and *k _2_* for factor “E” are 8.36 GPa, 8.36 GPa, 8.37 GPa, 8.51 GPa, and 8.24 GPa, respectively. It is thus determined that the optimal solution is A_1_B_3_C_1_D_4_E_2_.

Furthermore, the order of influence for the adhesive strength is as follows: NaAlO_2_ concentration > frequency > duty radio > voltage > treating time. The maximum value of R is 30.49 N, and the minimum value is 6.07 N for factors A and E, respectively. Additionally, the values of *k _1_* for factor “A”, the *k _3_* for factor “B”, *k _1_* for factor “C”, *k _4_* for factor “D” and *k _1_* for factor “E” are 96.76 N, 82.70 N, 90.72 N, 90.99 N, and 82.88 N, respectively, which corresponds to the highest average adhesive strength among the four levels of each factor. Therefore, the optimal solution is A_1_B_3_C_1_D_4_E_1_.

In conclusion, the treating time is the least influential factor, and the three optimization schemes yield disparate results solely with regard to this variable. The average CoF represents the most pivotal evaluation criterion for the wear resistance of the composite coatings. Consequently, the A_1_B_3_C_1_D_4_E_4_ optimization scheme is selected. In other words, the synthesized MAO/MoS_2_ composite coating exhibits the optimal overall wear resistance, as evidenced by the markedly low CoF as well as high microhardness and adhesive strength, when the NaAlO_2_ addition is 10 g/L, the voltage is 500 V, the current frequency is 200 Hz, the duty ratio is 20%, and the treating time is 30 min.

### 3.2. Comprehensive Wear Performance

Figure 1a–d depicts the friction coefficient plots of the composite coatings prepared at voltages of 450, 475, 500, and 525 V, respectively. In general, the CoF curves of the 1-1, 2-1, 3-1, and 4-1 specimens, which have the same NaAlO_2_ concentration of 10 g/L, correspond to the lowest values in each of the four plots. Moreover, the average CoF is less than 0.15, exhibiting minimal fluctuations. Subsequently, the CoF of the composite-coated specimens at 500 V voltage, as illustrated in Figure 1c, ranges from 0.08 to 0.45 and exhibits the lowest average value. These findings indicate that the optimal tribological performance of the composite coatings is achieved when the NaAlO_2_ concentration is 10 g/L and the voltage is 500 V, which is consistent with the optimal level identified in Section 3.1.

At a voltage of 450 V, it is observed that the CoF of specimens 5-1 and 13-1 exhibits pronounced changes and significant fluctuations after 40 min of steady friction. In contrast, the CoF curves of specimens 1-1 and 9-1 exhibit only minor alterations and minimal fluctuations. Upon increasing the voltage to 475 V, the CoF of specimens 10-1 and 14-1 is observed to persist for 37 min of steady friction, followed by a notable shift and substantial fluctuations. In contrast, the CoF curves of specimens 2-1 and 6-1 exhibit minimal fluctuations, with values of ~0.15 and 0.2, respectively. Upon further increasing the voltage to 500 V, it is observed that the CoF of specimens 7-1 and 11-1 exhibits a notable steepening, reaching ~0.44 and 0.37, respectively, after 70 min of friction. In contrast, the CoF curves of specimens 3-1 and 15-1 remain at the stable friction stage, exhibiting a value of ~0.13. Upon increasing the voltage to 525 V, it is observed that the CoF of specimens 8-1, 12-1, and 16-1 exhibits a gradual increase from 0.16 to 0.30 after 50 min of friction. Subsequently, a pronounced and erratic change is observed, while the CoF curve of specimen 4-1 exhibits prolonged fluctuations above and below 0.13. This pronounced shift in the CoF curve can be attributed to alterations in the contact area and the formation of wear debris or abrasive particles in the wear tracks [43,44], which diminish the coating’s wear resistance.

**Figure 2:**
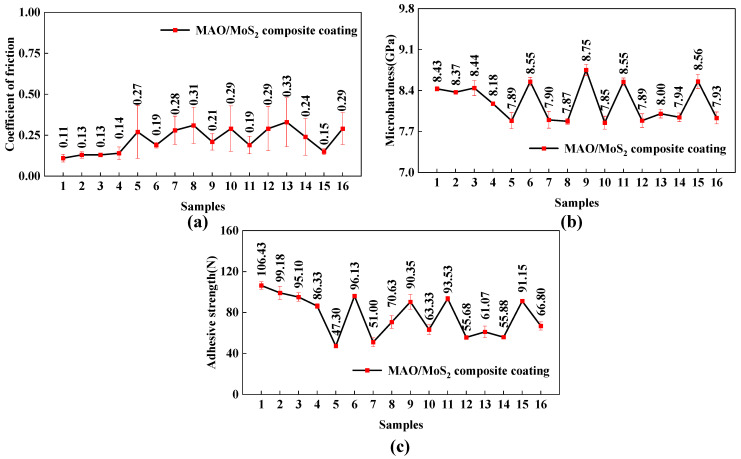
Comparison plots of composite-coated specimens at varying levels of wear resistance performance: average CoF (**a**), microhardness (**b**), adhesive strength (**c**).

Figure 2 presents a comparison of the average CoF, microhardness, and adhesive strength of the composite coatings, respectively. As demonstrated in Figure 2a, the average CoF values range from 0.10 to 0.34, with a boundary of 0.22 delineating the low and high CoF values. Furthermore, all specimens 1-1, 2-1, 3-1, 4-1, 6-1, 9-1, 11-1, and 15-1 are situated within the low CoF region, exhibiting narrow error bars in the comparison graph that align with the trend of slight increases illustrated in the friction coefficient curves observed in Figure 1. The remaining specimens are situated within the high CoF region, with a high frequency concentrated in the range of 0.26–0.30, and exhibit considerable error bars in the comparison graph. The graph is consistent with the observation that the friction coefficient curves in Figure 1 exhibit pronounced fluctuations and steep changes.

As illustrated in Figure 2b, the microhardness test results for the various composite coatings demonstrate that the microhardness values, ranging from 7.85 to 8.75 GPa with a boundary of 8.10 GPa, effectively differentiate between the low and high microhardness regions. The comparison graph indicates that, in comparison to specimens 1-1, 2-1, 3-1, 4-1, 6-1, 9-1, 11-1, and 15-1, which are in the high microhardness region, all other specimens are in the low microhardness region, with the microhardness concentrated in the range of 7.85–8.00 GPa. In relation to Figure 2a, it is determined that specimens within the high microhardness region exhibit diminished average CoF values. This outcome is attributed to the fact that the friction and wear behaviors of the coatings are highly sensitive to variations in their hardness [38].

Figure 2c illustrates the adhesive strength of the various composite coatings. The figure indicates that the adhesive strength spans a range of 47–107 N, which is divided into two distinct regions: weak binding and strong binding. The boundary strength is 77 N. The comparative plots reveal that specimens 1-1, 2-1, 3-1, 4-1, 6-1, 9-1, 11-1, and 15-1 exhibit robust bonding characteristics, with the adhesive strength centered in the 87–97 N region. In contrast, the remaining specimens exhibit weaker bonding properties. In relation to Figure 2a,b, it was determined that specimens exhibiting strong bonding exhibited both low average CoF and high hardness. Conversely, specimens with weak bonding demonstrated the opposite characteristics.

In conclusion, the 1-1, 2-1, 3-1, 4-1, 6-1, 9-1, 11-1, and 15-1 specimens demonstrate low average CoF value within the 0.10–0.22 range in tribological experiments, as well as high microhardness within the 8.18–8.75 GPa range and robust adhesive strength within the 82–107 N range. These findings indicate that the composite coatings prepared under the aforementioned eight groups of MAO process parameters exhibit exceptional comprehensive wear resistance properties. Thus, composite coatings with superior integrated wear resistance tend to have high surface microhardness and adhesive strength, ensuring the wear resistance of the coating [37]. The hardness variation directly affects the friction and wear behavior [38], and thus, the coatings also exhibit very low CoF and minimal fluctuations in dry friction experiments.

### 3.3. Optimized Wear Performance

Once the optimal level is selected, the next step is to prepare optimized specimens and test their wear resistance in order to verify the improvement in the wear resistance of the composite coatings resulting from the optimized NaAlO_2_ concentration and electrical parameters. The pertinent MAO process parameters are presented in Table 4.

**Figure 3:**
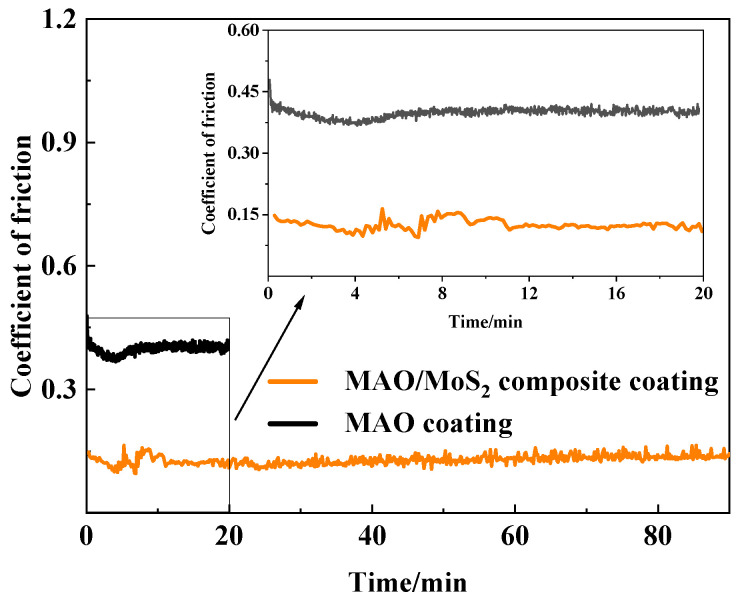
Plots of the friction coefficient and the local magnification of the optimized MAO and composite coatings.

Figure 3 depicts the CoF vs. wear time for the optimized MAO-coated specimen and the composite-coated specimen under an applied high load of 20 N. It can be observed that the variation of COF with wear time is attributable to the expansion in contact area and the accumulation of wear debris or abrasive particles in the wear tracks [43,44]. Initially, the optimized composite coating and the optimized MAO coating enter a running-in stage, with 11 and 4 min, respectively. During this stage, contaminants are removed and polished from the specimen and the grinding ball surface [30]. Following the running-in stage, the CoF of the optimized MAO coating attains a minimum value of 0.37. Subsequently, both specimens enter a steady-state stage, during which the CoF of both is ~0.12 and 0.40, respectively, continuously displaying a slow upward trend. Furthermore, as illustrated in the local magnified image, the CoF of the optimized composite coating displays fluctuations around 0.13 during the 20-min wear time. There is a notable reduction (~68%) in the average CoF compared to that of the optimized MAO coating, implying the lubrication effectiveness of the hydrothermal synthesis MoS_2_ layer strategy. Consequently, the composite coating exhibits a more significant friction reduction effect than the single MAO coating [19].

Table 5 presents a comparative analysis of the optimal wear resistance performance values of the composite specimens in optimized and orthogonal experimental conditions. The average CoF, microhardness, and adhesive strength values for the optimized composite-coated specimen, as presented in Table 5, are 0.13, 8.56 GPa, and 108.125 N, respectively. However, the values of the lowest average CoF, the highest microhardness, and the strongest adhesive strength in the orthogonal experimental are 0.11 (specimen 1-1), 8.75 GPa (specimen 9-1), and 106.43 N (specimen 1-1), respectively. Given that the microhardness of the 1-1 specimen is a mere 8.43 GPa, and the average CoF and adhesive strength of the 9-1 specimen are 0.21 and 90.35 N, respectively, it can be concluded that the combined wear-resistant performance of the two specimens is not as robust as that of the optimized composite coating. This confirms that the optimized composite-coated specimen exhibits the most comprehensive wear resistance performance and that the optimization effect is considerable.

### 3.4. Optimized Coating Properties

**Figure 4:**
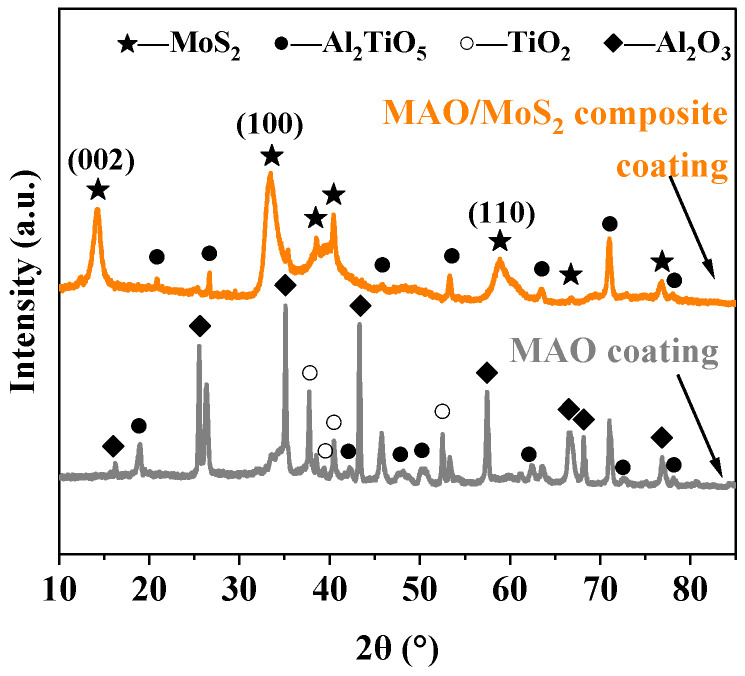
XRD patterns of optimized MAO coatings and composite coatings.

Figure 4 depicts the XRD patterns of the MAO coating and composite coating prepared under the optimized process parameters. The MAO coating is found to be primarily composed of Al_2_TiO_5_, Al_2_O_3_, and TiO_2_ as a result of the participation of ${AlO}_{2}^{-}$ in the film-forming reaction, which initially forms Al_2_O_3_. Subsequently, the secondary reaction in Equation (1) [45] occurs under the influence of the high temperature and high pressure of the plasma reaction:

(1) $${Al}_{2}O_{3}+{TiO}_{2}\to {Al}_{2}{TiO}_{5}$$

With regard to the MAO/MoS_2_ composite coating, the MoS_2_ diffraction peaks at 38.6° and 40.4° correspond to the rhombic 3R phase, and the distinctive strong diffraction peaks with sharp shapes align with the (002), (100), and (110) crystal planes of the hexagonal 2H-MoS_2_ peaks, thereby confirming that the primary material phase of the composite coating is MoS_2_. Furthermore, the XRD pattern displays faint Al_2_TiO_5_ peaks corresponding to the composition of the MAO coating. This phenomenon can be attributed to the limited thickness of the MoS_2_ self-lubricating coating, which is synthesized in-situ on the surface of the MAO coating. Consequently, the optimized composite coating is composed of a large quantity of MoS_2_ and contains a minor quantity of Al_2_TiO_5_.

**Figure 5:**
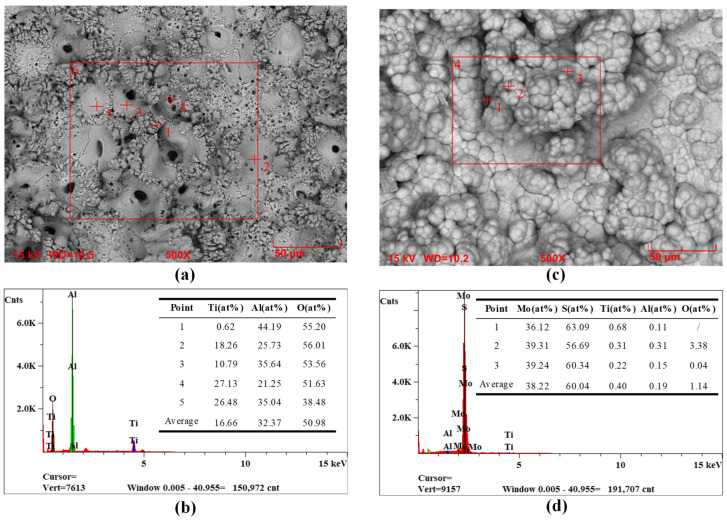
Surface morphology (**a**) and EDS analysis (**c**) of the optimized MAO coating; surface morphology (**b**) and EDS analysis (**d**) of the composite coating.

Figure 5 illustrates the surface morphology (Figure 5a,b) and the EDS analysis results (Figure 5c,d) of the optimized MAO coating and composite coating, respectively. As illustrated in Figure 5a, there are a multitude of substantial pores and microcracks on the MAO coating surface, as well as fragments or clustered particles in the vicinity of the micropores [46,47,48]. Given that the atomic percentage of aluminum (44.19%) and oxygen (55.20%) of point 1 in Figure 5c indicates the atomic number ratio close to 2:3, it can be inferred that the particle composition is predominantly Al_2_O_3_.

Furthermore, the mean atomic percentages of titanium and aluminum are 16.66% and 32.37%, respectively, with a ratio of approximately 1:2. This indicates that the primary product of the MAO reaction is Al_2_TiO_5_. Therefore, the MAO coating is composed primarily of Al_2_TiO_5_ and Al_2_O_3_, with a minor quantity of TiO_2_, consistent with the observed XRD pattern. The flowery globular structure observed on the composite coating surface in Figure 5b effectively fills the defects present in the MAO coating, thereby reducing the number of cracks and pores. This morphology is formed by the high-temperature curling of nanosheeted MoS_2_ during hydrothermal synthesis [29,49]. Subsequently, the EDS analysis of Figure 5d reveals that the atomic percentages of Ti, Al, and O on the surface are relatively low. In contrast, the atomic percentages of Mo and S are high, with average values of 38.22% and 60.04%, respectively. The ratio is close to 1:2, indicating that the hydrothermal products are primarily MoS_2_, consistent with the strong MoS_2_ peaks and the weak Al_2_TiO_5_ peaks observed in the XRD patterns.

**Figure 6:**
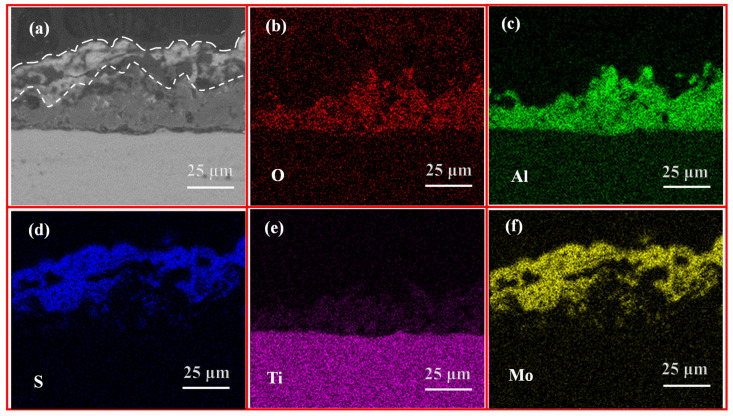
The cross-sectional morphology (**a**) of the optimized composite coating and the corresponding elemental mapping (**b**–**f**).

Figure 6 depicts the cross-sectional morphology (Figure 6a) of the optimized composite coating and the corresponding elemental mapping (Figure 6b–f). As illustrated in Figure 6, the MAO coating, comprising Ti, Al, and O, can be distinguished into a dense inner layer and a porous outer layer. Additionally, there is particulate Al_2_O_3_ on the surface. However, in the region where Ti, Al, and O are lacking, Mo and S are enriched, thereby filling the microporous interior of the MAO coating. This indicates that the hydrothermal reaction solution penetrates the pores and synthesizes MoS_2_ in situ, thereby reducing the pore defects. This is due to the porous outer coating of the MAO coating facilitating an increase in the mechanical interlocking [50], which in turn increases the bonding with the MoS_2_ coating [37]. Consequently, the composite coating displays a significantly enhanced adhesion compared to the MAO coating. In addition, the thicknesses of MAO layer and MoS_2_ layer are measured to be 27.84 ± 4.65 μm and 33.38 ± 7.09 μm, respectively.

### 3.5. Wear Resistance Analysis

**Figure 7:**
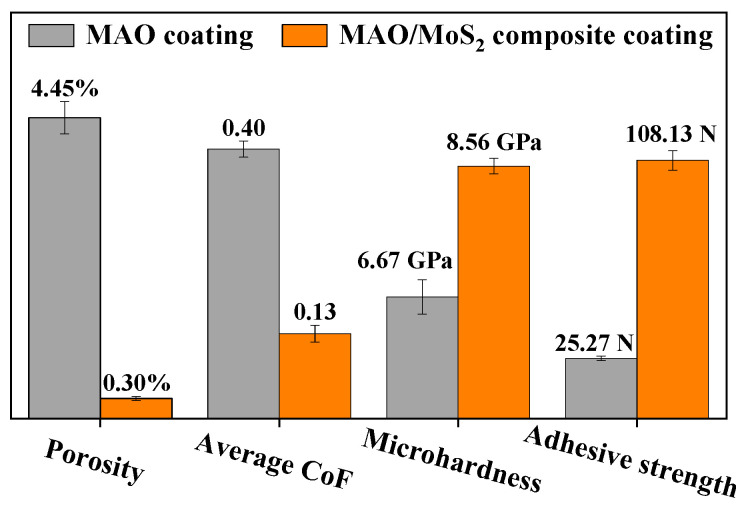
Comparison of porosity, average CoF, microhardness, and adhesive strength of optimized MAO coatings and composite coatings.

Figure 7 presents a visual comparison of the performance of of the optimized specimens in terms of porosity (calculated from Figure 5a,b), average CoF for a 20-min wear time, microhardness, and adhesive strength. It is observed that the porosity of the composite coating exhibits a reduction of 4.15% in comparison to the MAO coating, accompanied by a markedly diminished error bar. The outcome indicates that the surface becomes flatter after synthesizing the MoS_2_ layer, and it also provides evidence of its homogeneity, which is consistent with the micromorphology observed in Figure 5a,b. Subsequently, comparing the average CoF resulted in a reduction of 0.27 after the composite. Indicating the lubricating material MoS_2_ reduces friction, and enhances the tribological performance [19]. Following a comparison of the results of the microhardness test, it is determined that the microhardness of the composite coating increased by 1.90 GPa. As the change in hardness is significantly reflected in the tribological performance [34], an enhancement in the wear resistance of the coating can be expected. Finally, the test results for adhesive strength are compared, and it is found that the adhesive strength of the composite coating is enhanced by 82.86 N. This result is due to the porous microstructure of the MAO coating, which effectively increases the bonding with the MoS_2_ coating and also stores the MoS_2_ lubricant for friction, thus improving the wear resistance of the composite coating [37]. Consequently, the adhesive strength between the MoS_2_ self-lubricating layer and the MAO layer is augmented while the tribological properties are enhanced [51].

In conclusion, the optimized composite coating exhibits markedly enhanced wear resistance in comparison to the optimized MAO coating. The corresponding performances are as follows: there is a 93.3% reduction in porosity and a 58.2% reduction in average CoF, while there is a 43.2% increase in microhardness and a 327.9% increase in adhesive strength. From the perspective of coating porosity, this phenomenon can be attributed to the high porosity of the corresponding MAO coating, which provides an environment conducive to the growth of MoS_2_ lubricant material into a film on the porous outer surface. This results in enhanced bonding with the MoS_2_ coating [37], accompanied by a notable reduction in porosity and an increase in microhardness. The reduction in friction can be attributed to the flatter surface [52]. The coating is capable of maintaining a low CoF and minimal fluctuations for an extended duration under dry friction with a high load of 20 N. Consequently, the composite coating exhibits superior overall wear resistance.

Guo et al. [53] prepared MAO/MoS_2_ composite coatings on TC6 titanium alloy substrates by MAO process, followed by hydrothermal synthesis. The average CoF was ~0.42 in room temperature friction experiments at a friction distance of 90 m and a load of 5 N. In a separate study, Lv et al. [30] fabricated MAO/MoS_2_ composite coatings on 6063 aluminum alloy substrates in steps. The average CoF was ~0.2 in friction experiments at a load of 6 N and a friction distance of 222 m. A comparison of the present study with the previous two studies reveals that the TC4/MAO/MoS_2_ composite coating prepared in this paper exhibited an average CoF of ~0.13 in friction experiments at a high load of 20 N with a friction distance of 507 m at room temperature. This finding indicates that the coating demonstrated superior friction reduction performance.

## 4. Conclusions

In conclusion, MAO coatings are initially prepared on TC4 titanium alloy substrates and subsequently compounded into MAO/MoS_2_ self-lubricating composite coatings. Thereafter, an investigation is conducted into the composition, structure, morphology, and wear resistance of each coating, the principal findings of which are as follows:

1. The MAO/MoS_2_ composite coatings prepared under the eight groups of MAO process parameters (1#, 2#, 3#, 4#, 6#, 9#, 11#, and 15#) in orthogonal experiments exhibit a lower average CoF ranging from 0.10 to 0.22 over a 90-min wear period. Furthermore, the coatings display high microhardness and adhesive strength, with values ranging from 8.18 to 8.75 GPa and 82 to 107 N, respectively. These findings indicate that the composite coatings prepared using the aforementioned eight sets of parameters exhibit excellent comprehensive wear resistance.
2. The optimal process for MAO treatment on TC4 titanium alloy substrates is determined by an orthogonal experimental design. The process involves a NaAlO_2_ concentration of 10 g/L, a control voltage of 500 V, a frequency of 200 Hz, a duty cycle of 20%, and a treating time of 30 min. Following the optimization of the parameters, it is observed that the average CoF of the composite coating in the 20 N high-load dry friction experiments was ~68.5% lower than that of the MAO coating during the 20-min wear period. Following a 90-min wear period, a value of 0.13 is recorded. Additionally, the porosity of the composite coating was found to be ~93.3% lower, while the microhardness and adhesive strength exhibited increases of ~43.2% and 327.9%, respectively.
3. The optimized composite coating displays superior wear resistance in comparison to the MAO coating. This is attributed to the fact that the MAO coating possesses a porosity of 4.45%, which is characterized by the presence of numerous or large-size pores, as well as a multitude of particle clusters. Consequently, the hydrothermally synthesized MoS_2_ is capable of growing and filling the pores in situ, thereby forming a tightly attached lubrication film. The porosity of the composite coating is reduced to 0.30%, and its flat surface exhibits high microhardness (8.56 GPa) and adhesive strength (108.13 N). Additionally, the CoF value is as low as 0.13 with a gradual increase in 90-min wear time, indicating a notable enhancement in the wear resistance of the titanium alloy.

## Figures and Tables

**Table 1:** Factors and levels of the orthogonal experiment.

	Factors	A NaAlO_2_ Concentration (g/L)	B Voltage (V)	C Frequency (Hz)	D Duty Ratio (%)	E Time (min)
Levels						
1		10	450	200	5	15
2		15	475	300	10	20
3		20	500	400	15	25
4		25	525	500	20	30

**Table 2:** The orthogonal experiment Array and composite coating nomenclature.

	A	B	C	D	E	Composite Coating Samples
1#	10 (1)	450 (1)	200 (1)	5 (1)	15 (1)	1-1
2#	10 (1)	475 (2)	300 (2)	10 (2)	20 (2)	2-1
3#	10 (1)	500 (3)	400 (3)	15 (3)	25 (3)	3-1
4#	10 (1)	525 (4)	500 (4)	20 (4)	30 (4)	4-1
5#	15 (2)	450 (1)	300 (2)	15 (3)	30 (4)	5-1
6#	15 (2)	475 (2)	200 (1)	20 (4)	25 (3)	6-1
7#	15 (2)	500 (3)	500 (4)	5 (1)	20 (2)	7-1
8#	15 (2)	525 (4)	400 (3)	10 (2)	15 (1)	8-1
9#	20 (3)	450 (1)	400 (3)	20 (4)	20 (2)	9-1
10#	20 (3)	475 (2)	500 (4)	15 (3)	15 (1)	10-1
11#	20 (3)	500 (3)	200 (1)	10 (2)	30 (4)	11-1
12#	20 (3)	525 (4)	300 (2)	5 (1)	25 (3)	12-1
13#	25 (4)	450 (1)	500 (4)	10 (2)	25 (3)	13-1
14#	25 (4)	475 (2)	400 (3)	5 (1)	30 (4)	14-1
15#	25 (4)	500 (3)	300 (2)	20 (4)	15 (1)	15-1
16#	25 (4)	525 (4)	200 (1)	15 (3)	20 (2)	16-1

**Table 3:** Array and experimental results of the orthogonal experiment. (*k _i_* (*i* = 1, 2, 3, 4) and R represents the mean value of the corresponding level “*i*” for each factor and the extreme deviation, respectively).

	A	B	C	D	E	Average CoF	Microhardness (GPa)	Adhesive Strength (N)
1#	10 (1)	450 (1)	200 (1)	5 (1)	15 (1)	0.11	8.43	106.43
2#	10 (1)	475 (2)	300 (2)	10 (2)	20 (2)	0.13	8.37	99.18
3#	10 (1)	500 (3)	400 (3)	15 (3)	25 (3)	0.13	8.44	95.10
4#	10 (1)	525 (4)	500 (4)	20 (4)	30 (4)	0.14	8.18	86.33
5#	15 (2)	450 (1)	300 (2)	15 (3)	30 (4)	0.27	7.89	47.30
6#	15 (2)	475 (2)	200 (1)	20 (4)	25 (3)	0.19	8.55	96.13
7#	15 (2)	500 (3)	500 (4)	5 (1)	20 (2)	0.28	7.90	51.00
8#	15 (2)	525 (4)	400 (3)	10 (2)	15 (1)	0.31	7.87	70.63
9#	20 (3)	450 (1)	400 (3)	20 (4)	20 (2)	0.21	8.75	90.35
10#	20 (3)	475 (2)	500 (4)	15 (3)	15 (1)	0.29	7.85	63.33
11#	20 (3)	500 (3)	200 (1)	10 (2)	30 (4)	0.19	8.55	93.53
12#	20 (3)	525 (4)	300 (2)	5 (1)	25 (3)	0.29	7.89	55.68
13#	25 (4)	450 (1)	500 (4)	10 (2)	25 (3)	0.33	8.00	61.07
14#	25 (4)	475 (2)	400 (3)	5 (1)	30 (4)	0.24	7.94	55.88
15#	25 (4)	500 (3)	300 (2)	20 (4)	15 (1)	0.15	8.56	91.15
16#	25 (4)	525 (4)	200 (1)	15 (3)	20 (2)	0.29	7.93	66.80
*k _1_*_(CoF)_	0.13	0.23	0.19	0.23	0.21			
*k _1_*_(GPa)_	8.36	8.27	8.37	8.04	8.18			
*k _1_*_(N)_	96.76	76.29	90.72	67.24	82.88			
*k _2_*_(CoF)_	0.26	0.21	0.21	0.24	0.23			
*k _2_*_(GPa)_	8.05	8.18	8.17	8.20	8.24			
*k _2_*_(N)_	66.26	78.63	73.33	81.10	76.83			
*k _3_*_(CoF)_	0.24	0.19	0.22	0.24	0.23			
*k _3_*_(GPa)_	8.26	8.36	8.25	8.03	8.22			
*k _3_*_(N)_	75.72	82.70	77.99	68.13	76.99			
*k _4_*_(CoF)_	0.25	0.26	0.26	0.17	0.21			
*k _4_*_(GPa)_	8.11	7.97	7.98	8.51	8.14			
*k _4_*_(N)_	68.72	69.86	65.43	90.99	70.76			
R_(CoF)_	0.14	0.07	0.06	0.07	0.02			
R_(GPa)_	0.30	0.39	0.38	0.48	0.10			
R_(N)_	30.49	12.84	25.29	22.86	6.07			

**Table 4:** MAO process parameters and naming of optimized specimens.

NaAlO_2_ Concentration	Voltage	Frequency	Duty Ratio	Time
10 g/L	500 V	200 Hz	20%	30 min

**Table 5:** Optimization analysis for the orthogonal experiments.

	Orthogonal Experimental Optimum	Experimental A_1_B_3_C_1_D_4_E_4_
Average CoF	0.1067	0.1289
Microhardness	8.75 GPa	8.56 GPa
Adhesive strength	106.43 N	108.13 N

## Data Availability

The original contributions presented in the study are included in the article, further inquiries can be directed to the corresponding author.

## Abbreviations

The following abbreviations are used in this manuscript:

MAO	Micro-arc oxidation
CoF	Coefficient of friction
PTFE	Polytetrafluoroethylene
MAIP	Multi-arc ion plating
MoS_2_	Molybdenum disulfide
PPL	Para-polyphenylene
